# ChristianaCare Swank Memory Center: Launching GUIDE as a New Model Program

**DOI:** 10.1002/alz.095819

**Published:** 2025-01-09

**Authors:** Steven F Huege

**Affiliations:** ^1^ ChristianaCare Swank Memory Center, Wilmington, DE USA

## Abstract

N/A N/A N/A N/A N/A N/A N/A N/A N/A N/A N/A N/A N/A N/A N/A N/A N/A N/A N/A N/A N/A N/A N/A N/A N/A N/A N/A N/A N/A N/A N/A N/A N/A N/A N/A N/A N/A N/A N/A N/A N/A N/A N/A N/A N/A N/A N/A N/A N/A N/A N/A N/A N/A N/A N/A N/A N/A N/A N/A N/A N/A N/A N/A N/A N/A N/A N/A N/A N/A N/A N/A N/A N/A N/A N/A N/A N/A N/A N/A N/A N/A N/A N/A N/A N/A N/A N/A N/A N/A N/A N/A N/A N/A N/A N/A N/A N/A N/A N/A N/A N/A N/A N/A N/A N/A N/A N/A N/A N/A N/A N/A N/A N/A N/A N/A N/A N/A N/A N/A N/A N/A N/A N/A N/A N/A N/A N/A N/A N/A N/A N/A N/A N/A N/A N/A N/A N/A N/A N/A N/A N/A N/A N/A N/A N/A N/A N/A N/A N/A N/A N/A N/A N/A N/A N/A N/A N/A N/A N/A N/A N/A N/A N/A N/A N/A N/A N/A N/A N/A N/A N/A N/A N/A N/A N/A N/A N/A N/A N/A N/A N/A N/A N/A N/A N/A N/A N/A N/A N/A N/A N/A N/A N/A N/A N/A N/A

